# Orthopedic Professionals’ Recognition and Knowledge of Pain and Perceived Barriers to Optimal Pain Management at Five Hospitals

**DOI:** 10.3390/healthcare6030098

**Published:** 2018-08-13

**Authors:** Fadi Bouri, Walid El Ansari, Shady Mahmoud, Ahmed Elhessy, Abdulla Al-Ansari, Mohamed Al Ateeq Al-Dosari

**Affiliations:** 1 Department of Orthopedic Surgery, Hamad General Hospital, Hamad Medical Corporation, Doha 3050, Qatar; fadi.bouri@gmail.com (F.B.); dr_shady2006@yahoo.com (S.M.); AElhessy@hamad.qa (A.E.); maldosari1@hamad.qa (M.A.A.A.-D.); 2 Department of Surgery, Hamad General Hospital, Hamad Medical Corporation, Doha 3050, Qatar; AALANSARI1@hamad.qa; 3 College of Medicine, Qatar University, Doha 2713, Qatar

**Keywords:** orthopedic, inpatient pain, pain assessment/intensity, pain knowledge/management, pain attitudes, questionnaire, geriatric and pediatric pain

## Abstract

Pain is a challenge for orthopedic healthcare professionals (OHCP). However, pain studies examined the competencies of a single OHCP category, did not consider various pain management domains or barriers to optimal pain service, and are deficient across the Arabic Eastern Mediterranean region. We surveyed OHCP’s recognition and knowledge of pain and perceived barriers to optimal pain service (361 OHCP, five hospitals). Chi square compared doctors’ (*n* = 63) vs. nurses/physiotherapists’ (*n* = 187) views. In terms of pain recognition, more nurses had pain management training, confidently assessed pediatric/elderly pain, were aware of their departments’ pain protocols, and felt that their patients receive proper pain management. More doctors comfortably prescribed opiate medications and agreed that some nationalities were more sensitive to pain. For pain knowledge, more nurses felt patients are accurate in assessing their pain, vital signs are accurate in assessing children’s pain, children feel less pain because of nervous system immaturity, narcotics are not preferred due respiratory depression, and knew pre-emptive analgesia. As for barriers to optimal pain service, less nurses agreed about the lack of local policies/guidelines, knowledge, and skills; time to pre-medicate patients; knowledge about medications; complexity of the clinical environment; and physicians being not comfortable prescribing pain medication. We conclude that doctors required confidence in pain, especially pediatric and geriatric pain, using vital signs in assessing pain and narcotics use. Their most perceived barriers were lack of local policies/guidelines and skills. Nurses required more confidence in medications, caring for patients on narcotics, expressed fewer barriers than doctors, and the complexity of the clinical environment was their highest barrier. Educational programs with clinical application could improve OHCPs’ pain competencies/clinical practices in pain assessment and administration of analgesics.

## 1. Introduction

Healthcare seeks to decrease postoperative pain to ensure better and faster postoperative mobilization [1]. Postoperative pain pathways identify populations at risk for 30-day readmissions and emergency department visits that are not due to post discharge complications [2]. Chronic pain is acknowledged as a disease per se [3], and addressing pain control expectations before discharge could reduce surgical readmissions [2]. However, despite advances in perioperative protocols, pain remains a very frequent clinical symptom seen by orthopedic surgeons and a major reason for patients seeking medical assistance [3], and pain management after e.g., orthopedic procedures remains suboptimal for many patients [4].

For instance, 37% of orthopedic patients reported pain to be severe at its highest intensity [5], where postoperative pain remains a problem that requires consensus and joint efforts [5]. Similarly, patients feel moderate to severe pain after e.g., total knee arthroplasty [6], and managing such pain remains unsatisfactory [7]. Likewise, pain in elderly surgical patients is undermanaged, to the extent that the worst pain intensity in the first post-surgery day was the best predictor of patient satisfaction [8]. Moreover, the elderly population is increasing over time and is more likely to complain of painful conditions than other age groups [9]. Age was a major confounder of the sex differences in postoperative pain outcomes, especially in patients older than 50 years [10], and failure to manage pain in elderly populations might lead to serious adverse events [11]. Orthopedic patients are particularly less satisfied than other patients in terms of pain management [8].

In addition to pharmacologic pain control, non-pharmacologic means are employed to reduce pain e.g., preoperative education [8], sham “opioid” for placebo analgesia [12], mindfulness meditation pain relief [13], and other environmental methods [14]. Inadequate pain management results in negative clinical outcomes that influence patients’ responses and compliance to treatment [15,16]. Reasons for poor postoperative pain management are multifactorial and complex, e.g., inadequate opioid prescribing, failure to apply equianalgesic principles, fragmentation of care/lack of clear standards, and patient expectations. Whilst surgical pain guidelines exist, health practitioners do not consistently implement such guidelines [17,18,19], leading to deficient pain management by caregivers.

Optimal and effective pain management necessitates knowledgeable and trained healthcare practitioners, and appropriate attitudes and assessment skills [14,20]. For instance, high post-operative pain scores after “minor” orthopedic/trauma surgeries were partly attributed to inadequate prescription of opioid analgesics [21]. Elderly patients comprise a special concern, as the aging process affects the doses of opioid analgesics and contributes to failure of organs [22], and among older patients, postoperative pain management is a key element related to delirium [23].

In Turkey, pediatric surgical nurses needed more pain management education and more frequent use of different methods in care [14]. Likewise, nurses’ accurate assessment, appropriate intervention and evaluation of pain relief measures were critical for positive patient outcomes, where pediatric nurses had insufficient pain management knowledge and required education [24]. Certainly, pain control in orthopedic practice is challenging despite progresses in pain management [25], especially in elderly patients because of changes in their physiological responses to painful stimuli [26]. Adequate pain management is multi-disciplinary, where deficient staff education could impair such management [27]. Indeed, most healthcare professionals lack the ability to give effective pain control due to poor knowledge about drugs, doses, or side effects [28,29,30,31,32].

Whilst research exists on healthcare professionals’ pain management and knowledge, the literature reveals gaps. First, many studies examined the views/competencies of a single health professional category (nurses only) [14,20,24,33], omitting physicians’ views, knowledge, and skills, despite the fact that adequate pain management is multi-disciplinary [27], complex, and requires doctors and nurses to jointly implement therapeutic strategies [34]. Second, most studies did not consider the various pain management aspects holistically, resulting in piece meal investigations e.g., only focusing on health practitioners’ knowledge about either pain [14], recognizing pain [25], or pain management barriers [35]. Few studies addressed these multiple features collectively, e.g., knowledge and attitude in the same survey [33,36]. Third, research is deficient on the perceived barriers to optimal post-operative pain management, where a recent review [37] found only one study on such barriers [33], and advocated that such research is urgently needed. Fourth, geographically, pain management research is extremely lacking across the Eastern Mediterranean region, particularly in the Arab region [37], where, with the exception of Jordan [20,38], there were no studies from Arabian Gulf countries. 

Therefore, to bridge these gaps, the current study evaluated pain awareness in an Arabian Gulf country (Qatar). It surveyed the views of multiple health professionals (orthopedic surgeons including consultants, specialists, fellows and residents, and nurses and physiotherapists) across five hospitals and holistically examined the tripartite aspects of pain and its management, staff’s knowledge of pain and how they recognize it, and the barriers that limit optimal pain services.

The study’s objectives were to assess the levels of, and compare the responses of two categories of orthopedic health professionals (doctors vs. nurses and physiotherapists) in terms of their:

- Pain recognition abilities (e.g., training, confidence, awareness, prescribing, management);
- Knowledge of pain (e.g., assessing intensity, assessment methods, patient groups, narcotics and patients receiving narcotics, pre-emptive analgesia, non pharmacological);
- Perceived barriers to optimal pain service (e.g., policies and guidelines, monitoring, complexity, time to premedicate, indications/dosage knowledge); and
- Whether the total scores of each of the three questionnaire sections (pain recognition, knowledge of pain, perceived barriers) were correlated.

## 2. Materials and Methods

### 2.1. Ethics, Study Design, and Settings

The Medical Research Centre at the Hamad Medical Corporation (Doha, Qatar) (equivalent to the Ministry of Health) approved this cross-sectional study (IRB approved, Protocol 16177/16, 17 August 2016). Potential participants comprised all staff working at the Orthopedic Departments across four hospitals and the Bone and Joint Center. The participating institutions included those in Doha: Hamad General Hospital (largest hospital in Qatar, 603 beds, providing specialized and complex care) and the Bone and Joint Center (enhanced orthopedic specialist facility providing patient diagnostics and treatment, pain management, anesthesiologist and physiotherapy services), as well as the hospitals in the three main cities in Qatar: Alkhor General Hospital (Alkhor city, north Qatar, 115 beds), Alwakra Hospital (Alwakra city, south of Doha, 325 beds), and the Cuban Hospital (Dukhan city, west Qatar, 75 beds).

### 2.2. Procedures

All orthopedic healthcare professionals (surgeons, nurses, and physiotherapists, *N* = 361) at these five institutions were invited to participate in the survey. Potential participants were provided with information about the study aims and objectives, and if they agreed to participate, they verbally consented and completed a paper questionnaire. Participation was voluntary, the questionnaire was anonymous, and all data were confidential and protected. No incentives were provided for participation. A total of 253 completed questionnaires were collected from across the five participating institutions. Thus, the response rate was 70% (253 out of 361) using all orthopedic healthcare professionals in Qatar as the denominator, or 93.7% (253 out of 270) using the number of questionnaires distributed as the denominator.

### 2.3. Questionnaire

The survey questionnaire was adapted from published sources that assessed pain management for hospitalized orthopedic patients, healthcare providers’ knowledge and attitudes on pain, and pain knowledge among doctors and nurses [18,25,39,40,41,42,43,44]. The questionnaire collected demographic information of the health worker category/rank e.g., physician (resident, specialist/fellow, consultant), nurse or physiotherapist; place of work (Hamad General Hospital, Alwakra Hospital, Al Khor Hospital, Cuban Hospital, or Bone and Joint Center); and years in practice (<5 years, 5–10 years, >15 years). In order to undertake comparisons, we grouped together all physicians into a single group, and similarly grouped together the nurses and physiotherapists. 

The questionnaire comprised questions categorized into three sections comprising pain recognition; staff knowledge of pain; and obstacles and barriers to pain management items. The response format to all items was a five-point categorical scale (“Strongly Agree,” “Agree,” “Neither Agree nor Disagree,” “Strongly Disagree,” “Disagree”), later collapsed into three options for the analysis (“Strongly Agree/Agree,” “Neither Agree nor Disagree,” and “Strongly Disagree/Disagree”).

Pain recognition (nine items): tapped information on pain recognition and assessed the ability of the healthcare professionals to recognize the various aspects of pain. The items explored staff views on their formal training in pain management, confidence in assessing pain among patients of different ages (pediatric, elderly) or categories (trauma), awareness of pain scales/protocols and medications, patients’ receipt of proper pain management, sensitivity of different nationalities to pain, and the use of past information to write pain medication orders. 

Knowledge of pain (seven items): assessed participants’ medical knowledge of various aspects of pain. The items collected staff views on accuracy of patients’ pain assessment, assessment of pain and its levels in children, the use of the use of narcotics to control pain and the potential for addiction, pre-emptive analgesia, and non-pharmacological measures of pain alleviation. 

Perceived barriers to pain management (six items): asked participants about their views of the obstacles and barriers to pain management and assessed the difficulties that participants face regarding pain.

### 2.4. Statistical Analysis

Data were analyzed using the Statistical Package for Social Sciences (SPSS, version 17; Chicago, IL, USA), with significance level set at *p* < 0.05. The categorical responses of each item were compared (doctors vs. nurses) using Chi-square (χ^2^) test and presented as frequencies and percentages.

## 3. Results

### 3.1. Description of the Sample

The sample comprised 253 completed questionnaires from the five governmental hospitals across Qatar. It included 63 physicians (24.9%), 32 residents (12.8%), 23 specialists (9.2%), 8 consultants (3.2%)], 182 nurses (72.8%), and 5 physiotherapists (2%). Three questionnaires had missing information as to whether the respondent was a physician or otherwise. A sizeable portion of the respondents worked in institutions within Doha, and about one third of the sample had 5–10 years working experience (Table 1).

### 3.2. Pain Recognition

Table 2 shows that significantly bigger proportions of nurses (range 97.3–63%) than doctors (26.2–68.3%) agreed/strongly agreed that they received formal pain assessment/management training (92.5%), were confident in assessing pain in pediatric (66.9%) or elderly (90.4%) patients, were aware of pain scales/protocols used in their departments (97.3%), and that patients receive proper pain management in their department (90.9%). More nurses than doctors confidently assessed pain in trauma patients and felt that that when patients are admitted for a painful condition, they would ask them about what has helped their pain in the past and use the information to write orders for pain medication, but differences were non-significant. Conversely, a significantly higher proportion of doctors (88.9–80.6%) than nurses (69.5–63%) agreed/strongly agreed that they felt comfortable in prescribing pain medications, e.g., opiates, and that some nationalities are more sensitive to pain than others.

### 3.3. Knowledge of Pain

Table 3 depicts that significantly bigger proportion of nurses (range 97.3–44.3%) than doctors (63.9–11.1%) agreed/strongly agreed that: the most accurate tool in assessing patient’s pain intensity is the patient (97.3%), vital signs are always accurate in assessing pain intensity in children (74.5%), children feel less pain because of their nervous system immaturity (54.9%), narcotics use is not preferred because of their respiratory depression (61.8%), and that they were familiar with pre-emptive analgesia (44.3%). More nurses than doctors reported that a large percentage of patients receiving narcotics regularly become addicted, but differences were non-significant. Conversely, a significantly higher proportion of doctors (63.9%) than nurses (56.9%) agreed/strongly agreed that non-pharmacological measures can decrease pain perception.

### 3.4. Perceived Barriers to Optimal Pain Service

Table 4 depicts that significantly lower proportion of nurses (range 41.6–32.7%) than doctors (68.9–19.7%) agreed/strongly agreed that obstacles included lack of local policies/guidelines/knowledge and skills (39.3%), physicians not comfortable prescribing pain medication because of lack of proper monitoring (34.9%), complexity of clinical environment/lack of priority (41.6%), lack of time to premedicate patients before procedures (33.7%), lack of knowledge about indications/dosage of medications (e.g., Narcotics) (32.7%), and that the patient is NPO (32.7%).

Table 5 shows that whilst doctors had significantly higher scores across the pain recognition and knowledge of pain sections, nurses scored significantly higher in perceived barriers to pain recognition. In terms of location of the hospital, there were no significant differences between doctors’ and nurses’ knowledge of pain and perceived barriers to pain recognition. However, nurses employed in hospitals outside the capital city Doha scored higher in pain recognition. As for years of practice (experience), there were no doctor-nurse differences for pain recognition and knowledge of pain; but for the perceived obstacles/barriers to pain recognition, longer experience was associated with a steady significant rise in score. Table 5 also depicts the comparisons by the demographic parameters doctors’ vs. nurses’ responses, hospital location, and years of practice.

Table 6 shows that across the whole sample, knowledge of pain was significantly positively associated with pain recognition and was also significantly positively associated, albeit to a lesser extent, with the perceived obstacles/barriers to pain recognition.

## 4. Discussion

Understanding and managing pain are challenges to healthcare systems [45]. Whilst pain management has received attention [46], studies are deficient across the Arabic Eastern Mediterranean region [37], with none from the Arabian Gulf countries. This is despite shortfalls between clinician knowledge and its everyday usage in analgesic dosing/administration, non-pharmacological treatments, and pain assessment and management [46]. We assessed pain awareness of multiple health professionals in Qatar, focusing on pain management, knowledge of pain and its recognition, and perceived barriers to good pain service.

### 4.1. Pain Recognition

Most of the nurses (93%) agreed that they had formal pain management training, in accord with the USA, where all the nurses had pain management training [25], but in contrast with Saudi Arabia and others, where pain management was not emphasized in nurses’ education [47], or where <25% of nurses had recent pain management education [48]. Conversely, 69% of the doctors reported no formal pain management training, supporting others [25]. Whilst Cordts et al.’s [25] doctors comprised only residents (*n* = 20), the doctors’ sample in the current study comprised 51% residents, rendering both samples comparable. We also generally agree with Saudi Arabia [49], and although they did not use precisely the same questions as in the current study, 70% of their sample (doctors and nurses) had low pain knowledge, 31% had significant knowledge deficit on pain/pain management, and most doctors had high knowledge, while most nurses had low knowledge. Appropriate training, knowledge, and assessment of pain are key for healthcare providers, as it affects physical and emotional aspects of patients’ lives [49].

The majority (81%) of doctors were comfortable prescribing opioids, supporting others where most residents had adequate training in using opioids [25], in contrast with the USA, where physicians had misconceptions about opioid prescribing, possibly leading to inadequate prescribing/pain management [50]. However, fewer nurses (63%) felt comfortable prescribing opioids, contrasting with others where most nurses had no adequate training in using opioids [25]. Nurses require training, as education programs improve the knowledge of safe opioid prescribing/administration [51]. Such findings are important, as for longer term opioid therapy, identifying those patients that may possibly be at risk prior to start of therapy and those in whom problems develop during therapy are challenging [52].

Regarding managing elderly patients’ pain, 68% and 90% of the doctors and nurses, respectively, confidently managed it, contrasting with others [25], where most doctors and nurses had difficulties in dealing with elderly patients’ pain or where there was nurses’ knowledge deficit regarding older peoples’ pain and its management, especially for opioids [48]. This is despite the fact that the elderly are the fastest-growing segment of society and undergo surgery more frequently [53], older age significantly predicts inadequate pain control postoperatively [54], and successful postoperative pain management is critical for older surgical patients because pain affects perioperative outcomes [55]. However, given the pharmacokinetic and pharmacodynamic changes in older persons and the higher occurrence of co-morbidities and simultaneous use of other drugs, postoperative pain treatment must be cautiously adjusted for each patient [53].

In terms of sensitivity to pain, nurses (89%) and doctors (70%) felt that some nationalities are more sensitive to pain, supporting that pain sensitivity may differ among races/ethnicities, where African-American and Hispanic patients had lower pain tolerance than Caucasians [56,57,58]. Another perioperative issue is that, besides the varied sensitivity to pain, there also exists inter-individual variability in analgesic response and adverse effects of analgesic drugs, particularly for medications with narrow therapeutic indices e.g., opioids [59]. Sensitivity to pain might also be subject to age, where age factors had a direct association with pain [55], and a recent review [60] suggested that older age was associated with less postoperative pain, possible due to change in pain processing and pain modulation mechanisms [61]. 

### 4.2. Knowledge of Pain

It is concerning that many doctors were either neutral toward (43%) or disagreed with (24%) the statement that vital signs are always an accurate method to assess pain intensity in children, where others have confirmed such facts [14]. However, whilst non-pharmacological methods are important in decreasing pain perceptions [14], many of the nurses (64%) and doctors (57%) agreed to this true statement, supporting Italy, where most nurses (89%) and doctors (90%) correctly agreed that non-pharmacological distraction techniques decreased pain perception [43]. Nevertheless, it is concerning that our observed agreement levels to this statement were less than elsewhere [44], particularly as non-pharmacological techniques pivot clinicians away from dependence on medications to non-pharmacological pain treatment modalities [62]. 

In connection with children, 55% of the nurses agreed to the false statement that children feel less pain because of immaturity of their nervous system. Nevertheless, as in the current study, 47% of Turkish nurses answered the same question incorrectly [24]. In contrast, in Jordan, 65% of nurses disagreed to a similar false statement about children [20]. Incorrect nurses’ knowledge calls for educational programs, as children might suffer post-operative pain, where in addition to the lack of knowledge, pediatric nurses might use non-appropriate pain scales [63] and not record pain consistently [64].

As for the relationship between the use narcotics and respiratory depression, 62% of the nurses answered incorrectly, agreeing that narcotic use is not preferred because of respiratory depression (false statement). Our findings do not support Jordan, where 61% of nurses correctly answered the statement that respiratory depression rarely occurs in patients receiving stable doses of opioids over months (true statement) [20]. In terms of patients receiving narcotics regularly becoming addicted, 43% and 52% of the doctors and nurses, respectively, correctly disagreed to this false statement. Our findings support others [39], where 30% of healthcare providers correctly disagreed that 25% of patients receiving narcotics around the clock become addicted. Elsewhere [44], about half the doctors and nurses respectively disagreed to the exact statement. 

A majority of the doctors and nurses agreed that the most accurate tool to assess patient’s pain intensity is the patient himself (true statement). We support others [39], where 64% of healthcare providers agreed to such sentiments, and elsewhere [44], 69% and 65% of the doctors and nurses, respectively, agreed to the statement. Treatment of pain is complex, as pain is subjective, and it is difficult for patients to explain and for caregivers to ascertain and remedy [45]. In addition, the lack of efficient screening instruments for detecting some types of very common pain e.g., acute or subacute low back pain, necessitate that each patient should be assessed individually (Reito et al., 2018 [65]). Hence, health professionals should assess their conversations with their patients, where better patient–doctor interactions and adequate clarifications for patients about the results of their procedure can align expectations and increase patient satisfaction (Al-Mohrej et al., 2018 [66]). Likewise, some types of chronic pain (e.g., chronic knee pain) could require depression management, conservative pain management measures, and increasing pain coping skills (Ventura et al., 2018 [67]). 

### 4.3. Perceived Barriers to Optimal Pain Service

Many of the doctors (53%) agreed that causes of inappropriate pain management included the complex clinical environment and the lack of priority. Likewise, 43% of the nurses disagreed to this statement, in line with the UK, where 39% of nurses felt that lack of time was the most common barrier to proper pain management [35]. A barrier to improper pain control in the USA was fear of dealing with narcotics [30]. Whilst 48% of the doctors supported such sentiments, more of the nurses (58%) disagreed that the fear of prescribing opioid medications/lack of knowledge about their use and dosage are barriers. In Turkey [34], 76% and 61% of nurses disagreed that inadequate assessment of pain and that physicians’ lack of trust in the nurses’ pain assessment were barriers to pain management, respectively. Such findings resonate with the fact that 52% of the nurses in the current study disagreed to that physicians were uncomfortable prescribing pain medication because of lack of proper monitoring. 

As for the lack of knowledge and skills as an obstacle to appropriate pain management, 69% of the doctors agreed, but about half the nurses disagreed to this barrier, in partial agreement with Australia [68], where 60% of nurses felt an obstacle was the lack of appropriate knowledge that led to increased pain among patients. 

Research of nurses’ perceived barriers to pain management found that 69% of nurses felt that a barrier was the lack of pain management guidelines, whilst 73% and 51% disagreed that inadequate staff knowledge of pain management is a barrier respectively [34]. The current study partially supports such findings, despite that we had the lack of local policies and guidelines for pain management and lack of knowledge and skills as a single question, where 51% of nurses disagreed to it. In Saudi Arabia [69], pain knowledge was deficient among doctors and nurses, partially supporting that in the current study, where the doctors had good pain management knowledge, but the nurses lacked some such knowledge and required improvement. Methodological differences in the instruments that assess pain, and the ways responses are reported render comparisons across various studies difficult and imprecise, highlighting the need for standardized tools and reporting of pain management research for meaningful and accurate comparisons. An important point is that effective pain management is multi-disciplinary, as multidisciplinary approaches offered better results compared to a mono-disciplinary approach [70], and deficient staff education could impair such management [27]. Effectiveness of complex chronic pain management in a biopsychosocial context includes physical, mental and social outcomes, and interdisciplinary multimodal pain therapy for such patients comprises at least psychological and physiotherapeutic interventions [71]. 

### 4.4. Limitations

This study has limitations. Self-selection, sociability, and social desirability cannot be excluded. We did not explicitly ask about e.g., knowledge of drug adverse effects. More information about participants’ demographics, gender, and educational background/training would have been beneficial. A larger sample size than the current 11.85% from hospitals outside the capital Doha would have been useful, hence generalizations should be cautious. We grouped together all physicians (resident, specialist, fellow, consultant). A larger sample would have allowed exploring the views of each category. Nevertheless, the study has strengths: it is the first in Qatar and the Arab nations of the Arabian Gulf, and one of the very few Eastern Mediterranean region studies, to survey across five hospitals the views of multiple categories of health professionals, focusing holistically on pain and its management, knowledge and recognition of pain, and the barriers that limit good pain services. We are not aware of others who have conducted such an undertaking.

## 5. Conclusions

Differences existed between physicians’ and nurses’ pain knowledge and practices, pain recognition, and perceived barriers to optimal pain service. Doctors required more formal training in pain management, confidence in pediatric pain assessment, knowledge about the value of vital signs in assessing pain intensity, and the appropriate use of narcotics. Doctors viewed the lack of local policies/guidelines and lack of knowledge and skills as their highest perceived barriers to optimal pain service. Nurses required more assurance in prescribing pain medications/narcotics and in caring for patients receiving narcotics. Nurses generally expressed less perceived barriers to optimal pain service than doctors, with the complexity of clinical environment being their highest barrier. Educational programs with clinical application are required to develop medical professionals’ pain knowledge and clinical practices, and their clinical competencies of pain assessment and administration of analgesics.

## Figures and Tables

**Table 1:** Demographic and work-related characteristics of the sample.

Occupation *N* (%)		Place of Work *N* (%)		Years Practice *N* (%)		
Doctors	Nurses/Physiotherapists	Doha	Outside Doha	<5	5–10	>15
63 (24.9)	187 (73.9)	212 (83.8)	30 (11.85)	60 (23.7)	83 (32.8)	63 (24.9)

**Table 2:** Pain recognition among orthopedic doctors and nurses (*N* = 253).

Pain Recognition	SA/A	NA/D	SD/D	*p*
	*N* (%)	*N* (%)	*N* (%)	
Received formal training in pain assessment/management				<0.001
Doctors	16 (26.2)	3 (4.9)	42 (68.9)	
Nurses	173 (92.5)	2 (1.1)	12 (6.4)	
Confident in assessing pain in pediatric group				<0.001
Doctors	17 (27)	30 (47.6)	16 (25.4)	
Nurses	121 (66.9)	33 (18.2)	27 (14.9)	
Confident in assessing pain in trauma patients				0.179
Doctors	50 (80.6)	8 (12.9)	4 (6.5)	
Nurses	167 (89.3)	15 (8)	5 (2.7)	
Confident in assessing pain in elderly patients				<0.001
Doctors	43 (68.3)	15 (23.8)	5 (7.9)	
Nurses	169 (90.4)	14 (7.5)	4 (2.1)	
Aware of pain scales/protocols used in my department				<0.001
Doctors	43(68.3)	14(22.2)	6 (9.5)	
Nurses	182 (97.3)	2 (1.1)	3 (1.6)	
Feel comfortable prescribing pain medications e.g., Opiates				0.019
Doctors	50 (80.6)	8 (12.9)	4 (6.5)	
Nurses	109 (63)	28 (16.2)	36 (20.8)	
Patients receive proper pain management in our department				<0.001
Doctors	38 (60.3)	20 (31.7)	5 (7.9)	
Nurses	170 (90.9)	13 (7)	4 (2.1)	
Some nationalities are more sensitive to pain than others				0.007
Doctors	56 (88.9)	2(3.2)	5 (7.9)	
Nurses	130 (69.5)	8 (4.3)	49 (26.2)	
Patients admitted for painful condition/s are asked what has helped their pain in the past, and information used to write pain medication orders				0.071
Doctors	42 (67.7)	13 (21)	7 (11.3)	
Nurses	146 (81.1)	19 (10.6)	15 (8.3)	

SA/A: strongly agree/agree; NA/D: neither agree or disagree; SD/D: strongly disagree/disagree; bolded cells indicate statistical significance; for all items, a higher number (percentage) of “strongly agree/agree” responses denotes better pain recognition by healthcare professionals.

**Table 3:** Knowledge of pain among orthopedic doctors and nurses (*N* = 253).

Knowledge of Pain	SA/A	NA/D	SD/D	*p*
	*N* (%)	*N* (%)	*N* (%)	
Most accurate tool in assessing intensity of patient’s pain is patient himself (True)				0.002
Doctors	54 (85.7)	8 (12.7)	1 (1.6)	
Nurses	182 (97.3)	4 (2.1)	1 (0.5)	
Vital signs always accurate in assessing pain intensity in children (True)				<0.001
Doctors	21 (33.3)	27 (42.9)	15 (23.8)	
Nurses	137 (74.5)	33 (17.9)	14 (7.6)	
Children feel less pain because of immaturity of nervous system (False)				<0.001
Doctors	7 (11.1)	20 (31.7)	36 (57.1)	
Nurses	100 (54.9)	34 (18.7)	48 (26.4)	
Narcotics not preferred to be used because they cause respiratory depression (False)				<0.001
Doctors	16 (25.8)	17 (27.4)	29 (46.8)	
Nurses	115 (61.8)	46 (24.7)	25 (13.4)	
Am familiar with pre-emptive analgesia (neither true nor false)				0.005
Doctors	16 (25.4)	24 (38.1)	23 (36.5)	
Nurses	77 (44.3)	65 (37.4)	32 (18.4)	
Non-pharmacological measures can decrease perception of pain (True)				<0.001
Doctors	39 (63.9)	18 (29.5)	4 (6.6)	
Nurses	99 (56.9)	27 (15.5)	48 (27.6)	
Large percentage of patients receiving narcotics regularly becomes addicted (False)				0.166
Doctors	26 (42.6)	16 (26.2)	19 (31.1)	
Nurses	90 (52)	27 (15.6)	56 (32.4)	

SA/A: strongly agree/agree; NA/D: neither agree or disagree; SD/D: strongly disagree/disagree; bolded cells indicate statistical significance; for true items, higher number (percentage) of “strongly disagree/disagree” responses denotes better healthcare professionals’ knowledge of pain; for false items, higher number (percentage) of “strongly disagree/disagree” responses denotes better healthcare professionals’ knowledge of pain.

**Table 4:** Perceived obstacles/barriers to pain recognition among orthopedic doctors and nurses (*N* = 253).

Obstacles and Barriers to Pain Recognition	SA/A	NA/D	SD/D	*p*
	*N* (%)	*N* (%)	*N* (%)	
Lack of local policies and guidelines, lack of knowledge and skills				<0.001
Doctors	42 (68.9)	9 (14.8)	10 (16.4)	
Nurses	67 (39.3)	16 (9.5)	85 (50.6)	
Physicians not comfortable prescribing pain medication because of lack of proper monitoring				<0.001
Doctors	30 (49.2)	18 (29.5)	13 (21.3)	
Nurses	59 (34.9)	22 (13)	88 (52.1)	
Complexity of clinical environment and lack of priority				<0.001
Doctors	32 (53.3)	18 (30)	10 (16.7)	
Nurses	69 (41.6)	25 (15.1)	72 (43.4)	
Lack of time to premedicate patients before procedures				0.001
Doctors	25 (41)	19 (31.1)	17 (27.9)	
Nurses	56 (33.7)	23 (13.9)	87 (52.4)	
Lack of knowledge about indications/dosage of medications (e.g., Narcotics)				<0.001
Doctors	29 (47.5)	18 (29.5)	14 (23)	
Nurses	55 (32.7)	16 (9.5)	97 (57.7)	
Patient is NPO				0.018
Doctors	12 (19.7)	17 (27.9)	32 (52.5)	
Nurses	54 (32.7)	22 (13.3)	89 (53.9)	

SA/A: strongly agree/agree; NA/D: neither agree or disagree; SD/D: strongly disagree/disagree; NPO: Nil Per Os; bolded cells indicate statistical significance; for all items, higher number (percentage) of “strongly agree/agree” responses denotes higher perceived obstacles/barriers to pain recognition by healthcare professionals.

**Table 5:** Mean sum of scores for each questionnaire section by selected parameters.

Parameter	Pain Recognition	Knowledge of Pain	Perceived Obstacles/Barriers to Pain Recognition
	M (SD)	M (SD)	M (SD)
Personnel			
Doctors	13.70 (3.09)	11.90 (2.12)	10.75 (3.11)
Nurses + Physio *	11.23 (2.75)	12.34 (1.99)	12.57 (4.79)
*p*	<0.001	0.137	0.006
Hospital Location			
In Doha	11.55 (2.69)	12.20 (2.08)	12.14 (4.58)
Outside Doha	13.33 (4.43)	12.16 (1.91)	12.53 (3.96)
*p*	0.002	0.928	0.659
Years in Practice			
<5	12.57 (4.023)	12.43 (1.98)	10.42 (3.56)
5–10	11.73 (2.82)	12.49 (1.90)	12.66 (4.61)
>15	12.00 (2.156)	11.76 (2.00)	13.75 (4.38)
*p*	0.273	0.059	<0.001

M: mean; SD: standard deviation; * nurses and physiotherapists together; bolded cells indicate statistical significance; lower means signify better outcome; minimum and maximum scores: pain recognition (9–27), knowledge of pain (7–21), perceived obstacles (6–18).

**Table 6:** Correlation between total scores of each of the three questionnaire sections.

Questionnaire Section	Pain Recognition	Knowledge of Pain	Perceived Obstacles/Barriers to Pain Recognition
Pain recognition	1	0.057 *	0.003
Knowledge of pain		1	0.020 **
Obstacles/barriers to pain recognition			1

Pearson correlation; * *p* < 0.001; ** *p* = 0.002.
